# Characterization of Kaposi's Sarcoma-Associated Herpesvirus-Related Lymphomas by DNA Microarray Analysis

**DOI:** 10.4061/2011/726964

**Published:** 2011-11-20

**Authors:** Keiji Ueda, Eriko Ohsaki, Kazushi Nakano, Xin Zheng

**Affiliations:** Division of Virology, Department of Microbiology and Immunology, Osaka University Graduate School of Medicine, 2-2 Yamada-oka, Suita, Osaka 565-0871, Japan

## Abstract

Among herpesviruses, **γ**-herpesviruses are supposed to have typical oncogenic activities. Two human **γ**-herpesviruses, Epstein-Barr virus (EBV) and Kaposi's sarcoma-associated herpesvirus (KSHV), are putative etiologic agents for Burkitt lymphoma, nasopharyngeal carcinoma, and some cases of gastric cancers, and Kaposi's sarcoma, multicentric Castleman's disease, and primary effusion lymphoma (PEL) especially in AIDS setting for the latter case, respectively. Since such two viruses mentioned above are highly species specific, it has been quite difficult to prove their oncogenic activities in animal models. Nevertheless, the viral oncogenesis is epidemiologically and/or *in vitro* experimentally evident. This time, we investigated gene expression profiles of KSHV-oriented lymphoma cell lines, EBV-oriented lymphoma cell lines, and T-cell leukemia cell lines. Both KSHV and EBV cause a B-cell-originated lymphoma, but the gene expression profiles were typically classified. Furthermore, KSHV could govern gene expression profiles, although PELs are usually coinfected with KSHV and EBV.

## 1. Introduction

Several viruses could induce cancers in human beings. For examples, some papilloma viruses (PVs) should be etiologic agents for cervical cancers [1], hepatitis B virus (HBV) [2] and hepatitis C virus (HCV) [3] for hepatocellular carcinomas, human T-lymphotropic virus 1 (HTLV-1) for adult T-cell leukemia (ATL) [4], Epstein-Barr virus (EBV) for Burkitt lymphomas, nasopharyngeal carcinomas (NPCs), and some of gastric carcinomas [5, 6], and Kaposi's sarcoma-associated virus (KSHV) for Kaposi's sarcoma [7], primary effusion lymphomas (PELs), and multicentric Castleman's disease [8–13]. Recently, a newly identified polyomavirus, Merkel cell polyomavirus, is nominated as an etiologic agent for Merkel cell carcinoma [14]. These viruses have too narrow host ranges to meet Koch's principles, and, therefore, there are a lot of augments about it. Nevertheless, causation between the viral infection and the related cancer formation could be evident epidemiologically and *in vitro* experimentally. 

Chronic inflammation caused by these viruses should be important factors, but it is not forgettable to keep in our minds that such inflammation itself is primarily caused by the viral infection [17]. Except for HCV and HTLV-1, these oncogenic viruses are usually DNA viruses and establish persistent or latent infection [18, 19]. Of course, HCV also establishes persistent infection in the infected hepatocytes [3]. Parts of some viral genomes in case of DNA viruses are integrated into host genomes, even though the process is not included in the life cycles. Integration could play roles for oncogenesis as shown for retroviral oncogenesis, and; thus, integration of viral genomes leads to promoter insertion mechanism to activate putative cellular oncogenes and host genome fragility [20]. If viral oncogenes are integrated and expressed, the effect should be more direct.

*γ*-herpesviruses such as EBV and KSHV are DNA viruses and do not have the genome integration process in their life cycles and just present as episomes in the infected nuclei for lives after establishing latent infection, since their genomes replicate and are partitioned according to the host cell cycles by utilizing host cellular replication machinery [6, 9]. Thus, the genomes act as complete extra genomes. 

KSHV was found in Kaposi's sarcoma tissues with representational difference analysis (RDA) as the eighth human herpesvirus by Chang et al. [21]. The sequence analysis revealed that this virus is not a member of *γ*1 or lymphocryptoviruses, which includes EBV, but *γ*2 or rhadinoviruses, whose prototype is Herpesvirus saimiri [22]. Extensive studies about the relationship between the virus infection and the diseases have shown that this virus is a causative agent for Kaposi's sarcoma [7], primary effusion lymphomas (PELs), and multicentric Castleman's disease [8], most of which happen in acquired immunodeficiency syndrome setting [23]. As for KS, KSHV is usually present in all types of KS: classical, iatrogenic, and African endemic KS and human immunodeficiency virus-1 negative gay men with KS [24]. Thus, it is doubtless that KSHV is an etiologic agent for KS and two lymphoproliferative diseases such as PEL and MCD as various kinds of *γ*-herpesviruses are related to some cancer formation [19].

KSHV has two life cycles: lytic infection/reactivation and latent infection as known for all herpesviruses. Among eight human herpesviruses, only EBV and KSHV establish latency *in vitro* especially Burkitt lymphoma [25] cell lines and PEL cell lines, respectively. In the latency, the viruses express a limited number of genes and replicate according to host cell cycle. The replicated genomes are partitioned into daughter cells, and; thus, the same copy number of the viral episomes is maintained, though details of the mechanism remain to be elucidated [26]. 

In case of KSHV, the viral latency seems to be very important for the maintenance of PEL, since the loss of the viral episomes leads to PEL cell death. Furthermore, EBV and KSHV usually coinfected in PEL but EBV is frequently lost while establishing PEL cell lines [27]. It has been unable for us to find out or establish subclones of KSHV-negative PEL cell lines from the parental lines [28]. In contrast, an EBV-lost BL cell line has been established [29].

Recently, we investigated gene expression profiles of several PEL cell lines [30] TY1 [31], BCBL1 [32], and its derivative D90 [28], BC3 [27], BC1 [33], in order to know the characteristic gene expression to maintain the PEL cells, comparing with those of BL lines: Ramos, Daudi, BJAB, Raji, and Akata [34]. And including T-cell-originated lymphoma cell lines: Jurkat, Molt3, SupT1 and MT4, we tried to know common features leading to lymphoma formation. Among PEL cell lines, only BC1 is coinfected with KSHV and EBV. BL cell lines are usually infected with EBV except Ramos and BJAB in the lineups this time. MT4 contains integrated human T-cell leukemia virus 1 (HTLV-1) genomes. Typically, the gene expression profiles were classified into either B-cell-originated or T-cell-originated pattern. And KSHV-associated PEL and usually EBV-associated BL showed typical gene expression profiles, respectively. Even though there was only one PEL cell line infected both with KSHV and EBV, its gene expression profile was classified as a KSHV pattern, suggesting that KSHV might make stronger influence on gene expression in the infected cells. In this paper, we would like to discuss and review about gene expression profiles of KSHV-associated B-cell lymphoma or lymphoma-like disease, while mining new data from our DNA array analysis or comparing ours to the others.

## 2. Gene Expression Profiles of KSHV-Related Tumors

As mentioned above, there are three definite diseases caused by KSHV. They are KS, PEL, and MCD. Especially in AIDS setting, KSHV has a very tight link with these diseases, and that is the virulence of KSHV emerges under the condition. It seems to be meaningful to know gene expression profiles of tumors, since such gives us information about origin of tumor cells, mechanism of tumorigenesis, designs of treatment, and so on. And thus; several reports have been published [25].

### 2.1. Kaposi's Sarcoma

The cellular origin of the spindle cells of KS is poorly defined and could be originated from vascular endothelial cells and various kinds of cytokines, chemokines, and growth factors are expressed [35, 36]. A recent report has shown that KSHV reprograms transcription profiles from angiogenic to lymphatic ones by inducing *PROX1*, a master regulator of lymphatic development and downregulation of blood vascular genes, in infected human dermal microvascular endothelial cells (HDMECs) [37, 38]. KSHV induces LYVE-1, reelin, follistatin, and desmoplakin as well as *PROX1*. These findings suggest that KSHV infection should induce a comprehensive reprogramming of blood vascular endothelial cells (BECs) to adopt a lymphatic endothelial cells (LECs). In the tissues of KS, a kind of cytokine and interleukin-6 (IL-6), basic fibroblast growth factor (bFGF), tumor necrosis factor-*α* (TNF-*α*), oncostatin M, interferon-*γ* (IFN-*γ*), and so on storm happens. In *in vitro* KSHV infection study; however, IL-6, oncostatin M, TNF-*α*, and IFN-*γ* inductions were not induced. In contrast, tumor growth factor *β*1/*β*3 and TGFb R2, CCL5 [39], CCL8 (MCP-2) and CCR5, and angiopoietin-2 (ang-2) were induced. Though such differences might be dependent on differences from environment for preparation of samples, some factors could be synthesized and secreted from the other kinds of cell type, because KS is actually a mixture of various kinds of tissues [35]. KS is basically latently infected with KSHV, and, thus usually does not express KSHV lytic genes [40]. It is, however, possible that lytic cycle is turned on especially just upon the infection and some lytic genes such as viral IL-6 (vIL-6), viral chemokines (vMIP-I, vMIP-II, and vMIP-III), possible oncogenic genes, such as K1, viral G-protein-coupled receptor (vGPCR) are expressed transiently and make an effect on various kinds of cellular gene expression [37]. Though details about mechanism remain to be understood, replication and transcription activator (RTA), a viral immediate early gene and a key inducer of viral lytic replication, must be expressed for lytic replication cycle. RTA is an extremely strong transactivator and functions both in a sequence-specific and a nonspecific manner. RTA could induce critical cellular gene expression and make a direction to KS formation [41, 42]. 

### 2.2. Multicentric Castleman's Disease

KSHV causes two B-cell-originated lymphoproliferative diseases: MCD and PEL [16]. MCD is a polyclonal and a kind of reactive lymphoproliferative disorder characterized by KSHV-infected monotypic cytoplasmic IgM-*λ*-expressing plasmablasts residing primarily in the mantle zone, dissolution of the follicles, and prominent interfollicular vascular proliferation [7]. MCD cells resemble mature B cells, as they express the preplasma cell markers, IRF4 and BLIMP1, the memory B-cell marker CD27, OCT2, and Ki67, though they are negative for certain B-cell-associated marker such as Pax5, CD20, CD30, and CD138 (syndecan-1) [43]. MCD plasmablasts are reported not to show somatic hypermutation in their rearranged IgV genes [7]. KSHV might preferentially target IgM-*λ*-expressing native B cells and differentiate into plasmablasts bypassing the GC reaction, although not all MCDs are infected with KSHV. In MCD, EBV is rarely coinfected [44]. It seems quite an interesting story, since both of viruses can infect a B-cell lineage and EBV usually disseminates more than 90% human beings and probably preexist in B cells before KSHV enters. It is unclear and should be elucidated whether MCD does not emerge in the presence of EBV, or development of MCD excludes EBV from the cells. 

It has not been successful to observe lymphoproliferation *in vitro* by infecting KSHV with peripheral blood mononuclear cells as shown for EBV, though KSHV infects CD19^+^B cells and establishes latency therein [32, 45]. From a point of view of gene expression profiles, high level interleukin 6 (IL-6) expression is a well-known fact in MCD and should do something in MCD pathogenesis [46]. B-cell markers, CD20 and the memory B-cell marker CD27 are usually expressed, but B-cell activation markers such as CD23, CD38 and CD30 are not [43]. KSHV gene expression profiles are different from those in KS and PEL. It was reported that viral lytic genes, vIRF-1 and vIL-6, and ORF59 (a polymerase processivity factor, PF8) as well as a latent gene, LANA, were expressed, suggesting that not a few cells in MCD are in the lytic phase [47].

### 2.3. Primary Effusion Lymphoma (PEL)

PEL is a distinct subtype of non-Hodgkin's lymphoma associated with KSHV as mentioned. PEL most commonly presents with pleural, peritoneal, or pericardial malignant effusions without a contiguous tumor mass [16]. In contrast to MCD, PEL is usually coinfected with EBV in vivo, and; therefore, EBV could be involved in the onset of tumor formation. It could be likely that PEL with or without EBV is different from origin of B-cell differentiation state [48]. KSHV, however, is never lost when PEL is introduced into cell culture maintenance *in vitro*, even if EBV frequently is lost from PEL cell lines. Thus, there is a strong linkage between the existence of KSHV and the maintenance of PEL cell lines *in vitro*. PEL is thought to be originated from post-GC plasmablastic cells [49]. Both PEL and MCD have a plasmablastic phenotype but should be different in the terms whether they are post-GC or bypass-GC reaction, respectively, [50]. 

EBV is another human oncogenic *γ*-herpesvirus, a putative causative agent of BL, NPC, some gastric carcinoma, NK lymphoma, and so on [6]. BL is also originated from GC B-cell and known for *MYC-IgH* or *MYC-IgL* rearrangement [15, 51]. Study on gene expression profiles using *in vitro* infection systems showed that cyclin-dependent kinase inhibitor 1 (CDKN1A; CIP1/WAF1; p21, U09579), interleukin-15 receptor *α* subunit precursor (U31628), interferon-induced 56-kd protein (IFI-56 K, X31628), and protein-tyrosine phosphatase 1C (PTP1C) SHP1 (X62055) and HLA class II histocompatibility antigen *α* chain (K01171) and HLA-DR antigen-associated invariant subunit (X00497) were prominently induced by the factor of ten or more [52]. High-mobility group protein (HMG-I, M23619), proliferating cyclic nuclear antigen (PCNA, M15796), endonuclease III homolog I (U79718), poly(ADP-ribose) polymerase (PARP; PPOL, M18112), erythroblastosis virus oncogene homolog1 (ETS-1, J04101), p-GAP hematopoietic protein C1 (RGC1, X78817), and c-myc (V00568) were remarkably reduced by the factor of five to eight hundred [52]. In this paper, EBV was infected with EBV-negative BL cell lines. The infected cells showed latency III phenotype, which is corresponding to lymphoblastoid cell lines (LCLs) established by EBV infection to PBMC *in vitro*. Most of BL, however, show latency I phenotype, and thus; this experiment model reflects LCL rather than BL [6]. Another report also utilized an EBV-depleted BL cell line, EBV^−^ Akata [53]. EBV^−^ Akata and EBV^+^ Akata were stimulated with IgG crosslinking, and lytic replication was induced. They analyzed cellular gene expression as well as viral gene expression. In this case, data did not reflect effects of EBV on BL, since this was just lytic replication/reactivation process, and almost all viral genes were expressed, which presumably took a substantial effect on cellular gene expression.

We recently investigated gene expression profiles of PEL cell lines, comparing with those of the other uniquely categorized cell lines, one of which was BL cell lines with or without EBV infection and another of which was T-cell leukemia cell lines (TCLs) [30]. All PEL cell lines are infected with KSHV, and one of them, BC1, is coinfected with KSHV and EBV. BL cell lines are usually infected with EBV, but Ramos and BJAB are not infected with EBV. TCL cell lines are heterogeneous. Jurkat was established from an acute T-cell leukemia, and Molt-3 and SupT1 were from a respective T-lymphoblastic leukemia, and MT4 was from an adult T-cell leukemia. Thus, differentiation status may be different among lines. 

Our obtained results were that three kinds of lines were typically classified into respective groups. Although the results might reflect just differentiation status of these cell lines, KSHV would never be lost from the PEL cell lines and BC1 coinfected with KSHV, and EBV was classified into the PEL cell category, suggesting that KSHV should be more dominant in gene expression control. Among about thirty thousand genes analyzed this time, we could extract sixty-three genes typically higher in BL cell lines and also sixty genes predominantly higher in PEL cell lines. For example, CD79A (NM_001738) and B (NM_000626), which are components of B-cell receptor and contain cytoplasmic immunoreceptor tyrosine-based activation motifs were highly expressed in BL without doubt. Accordingly, BCR downstream signaling 1 (BRDG1, NM_012108) was also higher in BL cell lines. A mature B cell-marker, CD22 (NM_001771), was characteristically expressed in BL not in PEL. Among very highly expressing genes in PEL, we found methyl CpG-binding domain protein (MBD1, NM_015845), interleukin 2 receptor beta (IL2RB, NM_000878), and angiopoietin 1 (ANGPT1, NM_001146). Such gene expression in PEL might suggest cellular environment and pathophysiologic status in the patient bodies under immunosuppression due to AIDS established by human immunodeficiency virus 1 (HIV-1) and KSHV infection.

**Figure 1:**
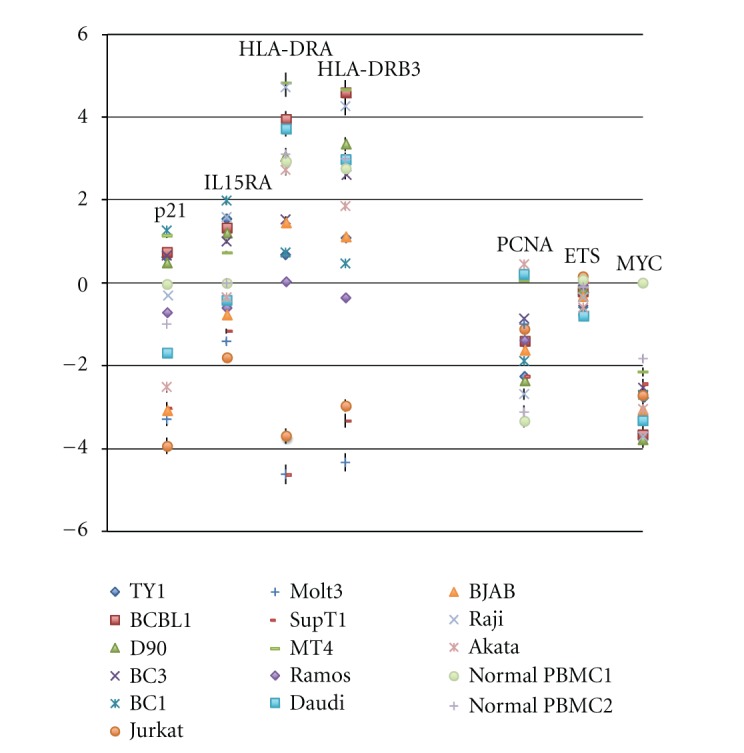
Genes increased or decreased in the presence of EBV [15] are picked up. The increased genes; p21^Cip1/WAF1^, IL15RA, HLA-DRA, and HLA-DRB3 were checked. The decreased genes: PCNA, ETS, and MYC (represented as N-myc in our case) were checked. Data are shown as log_2_ values with standard deviation. The concrete mean value of each gene expression was shown in Table 1.

Focused on p21^Cip1/WAF1^ (NM_000389), IL15 receptor *α* (NM_002189) and HLA-DR (HLA-DRA, NM_019111; HLA-DRB3, AF192259), which are reported to increase by EBV infection, this gene expression was indeed higher in B cell originated PEL and BL with a few exceptions in our analysis (Figure 1, Table 1). PCNA, ETS (L16464), and MYC (NM_005378) were relatively higher in TCL again with several exceptions, though MYC-Ig rearrangement was a feature of BL. 

**Figure 2:**
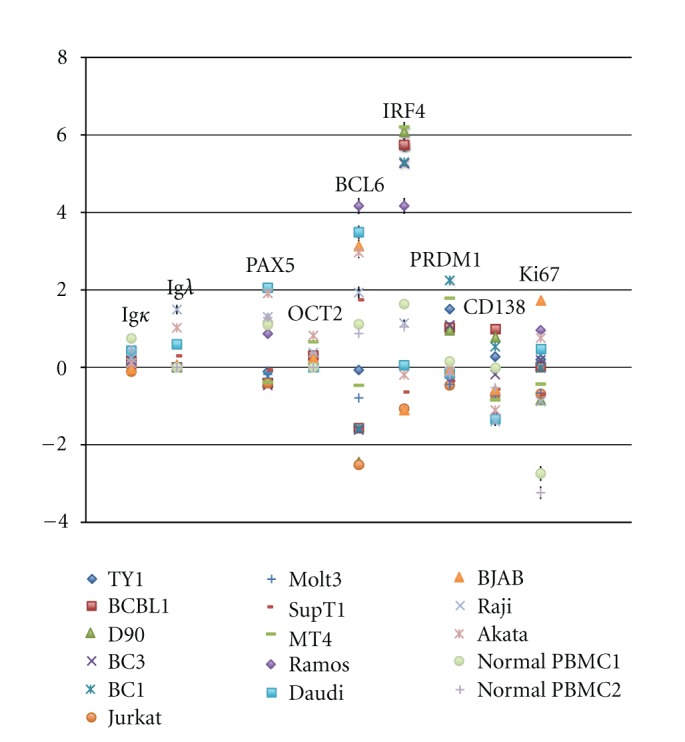
Genes characteristic in MCD are picked up. In MCD, Ig*κ*, PAX5, BCL6, CD138 are usually not expressed [16]. On the other hand, Ig*λ*, OCT2, IFR4/MUM1, PRDM1/BLINP1, and Ki67 are expressed [16]. Data are shown as log_2_ values with standard deviation. Characterization among PEL, BL, and TCL is shown in Table 2.

Paying attention to genes characteristic to MCD, a naïve B-cell marker: surface Ig lambda (XM_066332), B-cell specific markers: PAX 5 (NM_016734), Oct2 (XM_068123), and a GC B-cell marker: BCL6 (NM_001706) were higher in BL cell lines and preplasma cell markers; IRF4/MUM1 (NM_002460) and PRDM1/BLIMP1 (NM_001198) are definitely higher in PEL cells, assuring that BL should be derived from GC B cell and PEL from post-GC plasmablasts (Figure 2, Table 2). Plasma cell marker, CD138 (NM_002997), was also higher in PEL. Memory B-cell markers, Oct2 and Ki67 (NM_002417) expression, were not so different among three types of cell lines. Collectively, decisive differences between PEL and BL are low CD138 in PEL and high in BL, very low BCL6 in PEL, and very high in BL (Figure 2, Table 2). In addition, very strong expression of IRF4/MUM1 in PEL was characteristic, compared to the other cell lines.

**Figure 3:**
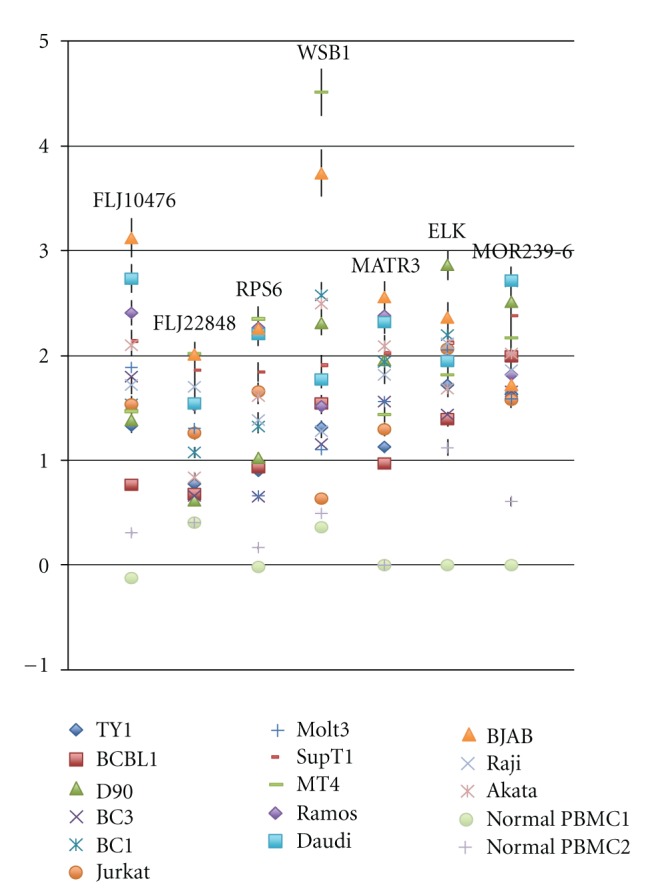
Highly expressing genes all in PEL, BL, and TCL are picked up. Data are shown as log_2_ values with standard deviation. Data are shown as log_2_ values with standard deviation. Detailed mean value of each gene is shown in Table 3.

If there are common genes in all tumor cell lines analyzed this time, such genes could be generally required for their establishment and/or maintenance. Thus, we mined the data in such point of view and found a couple of genes were commonly overexpressed compared to normal PBMC (Figure 3, Table 3). It is interesting that these include genes involved in signaling. However, since most of genes are not known well for their function, it remains to be clarified what they do and how important they are. 

In the same way, we also mined the data to find less expression in all types of cell line (Table 4), which might give disadvantage to cancer formation and/or maintenance. Actually, we found fifty or so of such genes, most of them are functionally unknown, and detailed analyses will be required in near future (data not shown). 

## 3. Conclusions

Studying gene expression profiles gives us various kinds of information. The analysis especially in cancer will lead to understanding how cancers are generated and maintained and to design what to do in order to suppress cancer growth. It is, however, just screening, and we have much work to do for this aim. 

## Figures and Tables

**Table 1:** 

																Normal	Normal
Name	ID	TY1	BCBL1	D90	BC3	BC1	Jurkat	Molt3	SupT1	MT4	Ramos	Daudi	BJAB	Raji	Akata	PBMC1	PBMC2
p21, Cip1 (CDKN1A), transcript variant 1	NM-000389	0.6168	0.7342	0.4951	0.6498	1.2574	−3.9304	−3.2876	−3.0266	1.1385	−0.7121	−1.6921	−3.071	−0.2815	−2.5181	−0.0306	−0.9993
																	
IL15RA transcript variant 1	NM-002189	1.5549	1.3211	1.2054	1.0005	1.9795	−1.7987	−1.4156	−1.1644	0.7295	−0.6013	−0.4221	−0.7741	1.5869	−0.3408	0	0
																	
HLA-DRA	NM-019111	0.6808	3.9458	3.0478	1.5288	0.7271	−3.696	−4.6163	−4.6238	4.8328	0.0282	3.7191	1.4575	4.7263	2.7318	2.9285	3.1007
																	
HLA-DRB3	AF192259	1.1026	4.5938	3.344	2.6277	0.4602	−2.9625	−4.33	−3.3242	4.657	−0.3435	2.9802	1.1189	4.2563	1.8549	2.7688	2.9922
																	
PCNA, transcript variant 1	NM-002592	−2.2538	−1.4012	−2.3533	−0.8696	−1.8885	−1.111	−1.0025	−2.2581	0.0462	−1.3735	0.2045	−1.6252	−2.6854	0.4512	−3.3202	−3.1112
																	
ETS oncogenes	L16464	−0.2124	−0.2223	−0.1314	−0.6449	−0.6913	0.1483	−0.1052	0.0186	−0.0974	−0.0638	−0.8105	−0.3384	−0.3456	−0.5868	0.0757	−0.1052
																	
MYCN	NM-005378	−2.7601	−3.6677	−3.7899	−2.5286	−2.7087	−2.7141	−3.2058	−2.4416	−2.1501	−3.3287	−3.329	−3.0585	−3.7126	−3.0375	0	−1.8303

**Table 2:** 

Lines	Gene						
	IRF4/MUM1	PRDM1/BLINP1	CD138	PAX5	BCL6	OCT2	Ki67
PEL	↑↑	↑	↑	↓	↓	→	→
BL	↑	→	↓	↑	↑↑	→	→
TCL	↓	↓	↓	↓	↓	→	↓

**Table 3:** 

Name	ID	TY1	BCBL1	D90	BC3	BC1	Jurkat	Molt3	SupT1	MT4	Ramos	Daudi	BJAB	Raji	Akata	Normal	Normal
																PBMC1	PBMC2
cDNA FLJ10476	AK001338	1.3263	0.7676	1.3801	1.7984	1.5367	1.5343	1.882	2.1362	1.4682	2.4062	2.7347	3.1228	1.7163	2.098	−0.1203	0.3065
																	
cDNA: FLJ22848	AK026501	0.7782	0.6786	0.6153	0.6563	1.0736	1.2594	1.3044	1.8615	2.0176	1.5196	1.5458	2.0081	1.7023	0.8386	0.4045	0.4004
																	
Ribosomal protein S6 (RPS6)	NM_001010	0.9017	0.9334	1.0244	0.6479	1.3239	1.6572	0.6572	1.8411	2.3493	2.2682	2.1999	2.2522	1.3873	1.6164	−0.0182	0.1647
																	
WD repeat and SOCS box-containing 1 (WSB1), transcript variant 1	NM_015626	1.3127	1.5445	2.3089	1.1516	2.5711	0.6377	1.0981	1.9078	4.5103	1.5161	1.7749	3.7406	1.2755	2.4918	0.359	0.4896
																	
Matrin 3 (MATR3)	NM_018834	1.1246	0.968	1.9522	1.5564	1.9376	1.2933	1.5574	2.0226	1.4332	2.3825	2.3212	2.5539	1.8178	2.0884	0	0
																	
ELK (LOC131341)	XM_067332	1.7184	1.391	2.8614	1.4346	2.1936	2.0576	2.0501	2.1184	1.813	1.9541	1.9471	2.3651	2.1104	1.6868	0	1.121
																	
Similar to olfactory receptor MOR239-6	XM_372502	1.6318	1.9947	2.5101	1.6554	2.706	1.5783	1.5849	2.3774	2.1667	1.8178	2.711	1.7203	1.8556	2.0178	0	0.603

**Table 4:** 

Name	ID	TY1	BCBL1	D90	BC3	BC1	Jurkat	Molt3	SupT1	MT4	Ramos	Daudi	BJAB	Raji	Akata	Normal	Normal
																PBMC1	PBMC2
cDNA FLJ20118	AK000125	−2.3823	−1.9072	−1.9797	−1.8911	−2.6889	0	−2.1411	−3.2798	−2.5236	−2.1555	−2.0963	−2.7591	−1.9995	−3.0018	0	0
cDNA FLJ12884	AK022946	−2.8453	−2.4261	0	−2.737	−2.4877	−2.3244	−0.8999	−1.0235	−1.4208	−2.4356	−3.054	−3.0835	−2.7217	−2.5435	0	0
cDNA FLJ13038	AK023100	−2.9994	−2.4772	−3.0186	−3.3026	−2.7287	−3.3596	−0.9996	−0.183	−2.7834	−2.658	−2.4415	−2.4291	−2.2883	−2.1052	0.3132	0.1399
cDNA FLJ13209	AK023271	−2.9254	−3.6178	−3.8287	−3.3465	−2.8999	−2.3812	−4.33	−2.8324	−3.6977	−3.658	−3.481	−3.5892	−3.4616	−3.7744	0	−0.5065
cDNA FLJ14567	AK027473	−3.3823	−3.6178	−2.7058	−1.8379	−3.0182	−1.3668	−2.0374	−2.2945	−1.1757	−3.073	−2.6242	−3.8415	−3.3297	−2.4208	0	−1.7585
cDNA FLJ31353	AK055915	−3.5001	−4.7198	−4.1118	−3.7967	−3.6791	−2.7417	−3.8866	−2.9092	−4.0524	−3.0009	−2.66	−2.2996	−1.3509	−4.1115	−0.3823	−0.1246
cDNA FLJ37955	AK095274	−1.2998	−0.1113	−1.5633	−1.317	−0.7984	−2.5666	−1.8192	−1.5649	−0.9224	−1.412	−2.5134	−2.0463	−2.5324	−1.4445	0	0
Odz3	AK125869	−2.0213	−2.4624	−1.4756	−1.3957	0	−2.9199	−2.5731	0	−2.6255	−2.2639	−2.4891	−2.0586	−2.4616	−2.5606	0	−1.0642
COL1A2	NM_000089_(2)	−3.44	−3.3222	−4.0878	−4.1915	−2.749	−3.696	−3.9297	0	−3.7933	−3.1489	−3.2724	−3.3744	−3.2883	−3.7349	0	0
Cystatin C (CST3)	NM_000099	−2.4327	−4.907	−6.1118	−1.2356	−5.4965	−2.5502	−3.4042	−3.4663	−1.4284	−5.6953	−6.1657	−2.9177	−5.7681	−5.5523	0.8547	1.1031
HBG2	NM_000184	−3.0213	−3.5694	−5.6968	−4.589	−3.1337	−4.9735	−5.8868	−4.6997	−5.983	−4.989	−5.0186	−4.4372	−6.0162	−5.7744	2.1347	0.3396
LYZ	NM_000239	−5.6627	−5.492	−6.0191	−3.9692	−5.5666	−6.0181	−5.1158	−5.3856	−5.3393	−5.8556	−4.8031	−4.7591	−5.3724	−4.9903	3.8701	4.0167
Serine (or cysteine) proteinase inhibitor	NM_000295	−5.2996	−1.4884	−4.5925	−5.7768	−5.245	−5.9302	−6.0676	−5.5169	−5.8128	−5.4198	−5.973	−5.8415	−5.7681	−5.5523	−1.0957	−1.0244
Interleukin 8 (IL8)	NM_000584	−3.066	−2.2442	−1.7518	−3.2322	−2.2886	−1.9093	−1.5561	−2.3167	−1.0642	−0.786	−2.1655	−2.3976	−1.9719	−0.8359	0.2761	1.0034
Interleukin 8 (IL8)	NM_000584_(2)	−4.5312	−3.1113	−3.6435	−2.4952	−3.6218	−3.7141	−3.7453	−2.7393	−2.7447	−4.3286	−4.4183	−2.8521	−3.5085	−3.4525	0.6967	1.5545
ANXA1	NM_000700	−1.6884	−2.7462	−5.7704	−4.139	−2.1007	−5.5584	−1.2667	−0.7569	−1.2654	−5.1886	−5.8851	−5.7587	−5.6238	−5.3593	−0.1914	−0.0244
TNFRSF1B	NM_001066	−0.0646	0.4257	−0.2587	1.098	0.1654	0.2687	0.0731	0.1623	0.208	−0.3584	−0.6991	0.0238	0.6067	−0.1162	2.0232	2.1586
ANPEP	NM_001150	−2.4773	−1.7507	−2.6092	−2.7866	−2.7593	−3.0066	−3.0552	−2.458	−2.3106	−1.6907	−2.3004	−2.5452	−2.1459	−2.0679	0.5172	0.5893
APOC1	NM_001645	−3.6284	−3.538	−2.7898	−3.554	−3.8226	−3.9735	−4.6522	−3.2088	−3.1376	−3.814	−4.0902	−3.9982	−3.6943	−4.0374	0	0
CRYAB	NM_001885	−1.994	−2.3767	−2.7519	−2.8275	−1.1204	−1.4943	−2.7645	−1.347	−1.245	−1.2816	−2.2518	−1.4818	−2.3089	−1.4287	0	0
GZMK	NM_002104	−3.7509	−1.7642	−3.2504	−2.8275	−3.0557	−3.9954	−3.6702	−3.6613	−2.8032	−2.2499	−3.3004	−2.2636	−2.2547	−2.2233	0.5673	0.6936
JUN	NM_002228	−2.2409	−2.7734	−3.1238	−0.3842	−1.8718	−0.968	−0.4387	−0.1644	−0.0479	−0.1719	−4.1148	−2.9517	−2.454	−3.7744	1.5962	0.4284
CD73 (NT5E)	NM_002526	−3.5469	−3.5694	−2.9525	−2.1262	−4.3968	−2.9955	−3.0552	−3.4173	−3.0405	−4.162	−3.53	−2.5027	−3.4164	−3.0495	0	0
PLAU	NM_002658	−1.32	−1.3256	−1.0046	−1.9922	0	−1.7095	−2.8448	0	0	−1.564	−2.6154	−2.3216	−2.005	−2.4366	0	0
CCL4	NM_002984	−4.0775	−3.0884	−3.2772	−3.6609	−3.8663	−4.0179	−4.2461	−3.7799	−3.2966	−3.8773	−3.1025	−3.0341	−0.0679	−2.7947	−0.2159	0.5398
ADAM12	NM_003474	−3.2734	−2.4118	−2.4872	−3.9021	−3.1744	−2.7141	−2.8448	−4.1018	−1.7494	−1.4316	−2.245	−2.8415	−2.3438	−1.2267	0	0
CST7	NM_003650	−4.8452	−3.9896	−4.0644	−4.9018	−4.4624	−2.9199	−2.9627	−3.2088	−3.7163	−3.0126	−3.7061	−3.8629	−3.59	−3.1371	−0.4389	−0.3123
Transmembrane protein with EGF-like	NM_003692_(2)	−2.671	−2.4047	0	−2.7765	−2.8225	−0.806	−1.1992	−1.3585	−2.3392	−2.3888	−2.642	−2.2147	0.3012	−2.8359	0	0
SEMA5A	NM_003966	−2.8649	−1.8483	0	−2.4386	−2.8885	−2.9304	−3.0552	−2.5429	−1.7116	−0.949	−2.1785	−2.5281	−1.9556	−1.4326	0	0
GNG11	NM_004126	−4.0104	−3.4189	−4.3748	−3.2049	−4.0558	−3.696	−3.9517	−4.0512	−3.2966	−4.0608	−3.9956	−2.2285	−3.0276	−3.535	0.048	0.2964
GZMB	NM_004131	−2.8163	−2.7373	−3.1483	−2.4788	−2.5577	−2.6085	−2.9627	−3.141	−2.1376	−2.5947	−3.4031	−3.0585	−2.8869	−2.5097	−0.3744	0.161
CSPG2	NM_004385	−2.8949	−2.3492	−3.3043	−2.6702	−3.2887	−3.3739	−3.4663	−3.6804	−1.2017	−3.0009	−3.4493	−2.8846	−2.8665	−3.4849	2.5309	2.5699
DUSP1	NM_004417	−2.0379	−0.9817	−1.9471	−1.6749	−1.8718	−1.5061	−0.9489	−1.1083	−0.0928	−0.8586	−2.0841	−0.4291	−1.1583	−1.8891	3.5758	3.3222
HRG-gamma	NM_004495	−3.0435	−2.7642	−3.5591	−3.3918	−3.365	−3.0066	−3.6702	−3.2088	−2.5484	−2.8349	−3.1273	−2.8415	−3.5733	−2.6315	0	0
PARG1	NM_004815	−3.579	−2.8197	−3.3321	−3.4545	−3.6218	−2.678	−2.6794	−3.0388	−3.2966	−2.0305	−3.9068	−3.4694	−3.1961	−2.4445	−1.0763	0
MYCN	NM_005378	−2.7601	−3.6677	−3.7899	−2.5286	−2.7087	−2.7141	−3.2058	−2.4416	−2.1501	−3.3287	−3.329	−3.0585	−3.7126	−3.0375	0	−1.8303
S100A11	NM_005620	−0.7903	−1.5457	−0.8165	−0.6245	−3.1072	−6.1108	−3.2326	−1.5212	−0.8772	−4.9434	−5.8851	−4.0462	−5.5571	−5.0866	−0.621	−0.388
S100A12	NM_005621	−1.8649	−2.5536	−2.3462	−3.3918	−2.2447	−1.863	−2.6611	−3.1148	−1.8131	−1.5468	−2.0481	−2.8415	−2.4389	−1.9388	3.5746	3.8939
SH3BP4	NM_014521	−2.0548	−2.5457	−2.7802	−3.2185	−1.8551	−2.374	−2.9853	−2.6997	−2.8641	−2.6396	−3.1149	−2.7391	−2.5244	−2.7546	0	0
SAMHD1	NM_015474	−1.7555	−2.2633	−2.2307	−2.1326	−2.3417	−3.9735	−4.2737	−3.4173	−2.4362	−1.7002	0.468	−2.6528	−1.6283	−0.3059	1.9357	2.0445
RAI14	NM_015577	−3.1478	−3.5225	−3.1857	−3.1135	−2.6889	0	−3.9079	−2.8007	−1.417	−1.3361	−2.9729	−3.5367	−2.7684	−3.9443	0	0
SH2D4A	NM_022071	−2.0049	−0.9299	−0.4187	−2.8589	−1.6987	−2.111	−2.3374	−1.8217	−1.4284	−0.7936	−1.9068	−2.0161	−1.4771	−1.5014	0	0
MATN2, transcript variant 2	NM_030583	−1.8949	−2.8873	−2.4563	−2.5976	−2.531	−3.1598	−2.5561	−2.2653	−2.4517	−2.2923	−3.1528	−2.5195	−2.4616	−3.4849	0	0
PTPNS1	NM_080792	−2.8748	−1.5575	−3.6787	−2.5118	−2.9697	−1.5791	−2.4194	−2.0954	−1.8081	−2.4922	−2.66	−2.0834	−2.4771	−2.0926	0.6197	0.1952
Similar to TCR delta chain (LOC122700)	XM_058650	−3.1358	−2.0828	−3.0413	−3.2459	−3.0684	−2.9199	−2.7452	−2.9903	−2.3832	−0.412	−2.803	−2.6161	−2.3368	−0.9728	1.3643	1.7152
